# Social survey on knowledge, attitude and practice of hypertrophic cardiomyopathy

**DOI:** 10.3389/fcvm.2025.1652963

**Published:** 2026-01-05

**Authors:** Weidong Lan, Bitong He, Sailing Hu

**Affiliations:** 1 Department of Rehabilitation Medicine, The First People’s Hospital of Xiaoshan District, Xiaoshan Affiliated Hospital of Wenzhou Medical University, Xiaoshan, China; 2 Department of Cardiology, Lishui Municipal Central Hospital and the Fifth Affiliated Hospital of Wenzhou Medical University, Lishui, China; 3 Department of Cardiology, Lishui Municipal Central Hospital and the Fifth Affiliated Hospital of Wenzhou Medical University, Lishui, China

**Keywords:** hypertrophic cardiomyopathy, social survey, knowledge, attitude, behavior

## Abstract

**Purpose:**

This study aimed to evaluate the knowledge, attitudes, and behaviors of patients with hypertrophic cardiomyopathy during the rehabilitation process and to explore the variables that influence these factors.

**Method:**

This study used a convenient sampling method to select 193 patients with hypertrophic cardiomyopathy who were treated in our hospital from June 2022 to 2024 as the research subjects. A special hypertrophic cardiomyopathy rehabilitation knowledge, attitude and behavior questionnaire was used to investigate the patients' rehabilitation cognition, emotional tendency and practical behavior. Then, univariate analysis and multivariate linear regression models were used to explore the key factors affecting knowledge acquisition, attitude formation and behavioral performance in the rehabilitation process of patients with hypertrophic cardiomyopathy.

**Result:**

The knowledge score of patients with hypertrophic cardiomyopathy was (7.91 ± 3.14) points, the belief score was (34.73 ± 3.16) points, the behavior score was (39.44 ± 3.95) points, and the total score was (82.08 ± 7.84) points. The study found that education level, work status and monthly per capita income were important factors affecting the knowledge, beliefs and behavior patterns of HCM patients (*P* < 0.05).

**Conclusion:**

There is room for improvement in the rehabilitation knowledge, beliefs and behaviors of patients with hypertrophic cardiomyopathy. In clinical practice, targeted intervention strategies should be designed and implemented based on the current rehabilitation cognition, attitude, behavior status and related influencing factors of such patients to optimize their rehabilitation cognition, attitude and behavior, thereby improving their quality of life.

## 1 Introduction

Hypertrophic cardiomyopathy (HCM), a common autosomal inherited heart disease caused by sarcomeric gene mutations, is characterized by hypertrophy of the left ventricle with varying morphology (1, 2). The disease is associated with a higher risk of atrial fibrillation and can manifest as atrial or ventricular arrhythmias, leading to sudden cardiac death, heart failure, and thromboembolic complications (3, 4). The natural history and cardiac structure of HCM show significant heterogeneity, resulting in diverse treatment responses (5, 6). Although with the advancement of medical technology, HCM has become a highly treatable disease and the mortality rate has been significantly reduced, there are still many unmet needs in its treatment field (7). The Knowledge, Attitude, Practice (KAP) survey, as a research tool to assess a specific group's understanding, attitude and behavior on a certain topic, is particularly important in health literacy research. It is based on the premise that knowledge can positively shape attitudes and ultimately influence behavior (8–10) (see Table 1 for details). Given the complexity and adverse consequences of HCM, as well as the relative paucity of current research on KAP status in HCM patients, it is particularly critical to explore the knowledge, attitudes, and behaviors of individuals affected by HCM. This study aims to provide a theoretical basis for health education for such patients by analyzing the current status of KAP and its influencing factors in 193 HCM patients.

**Table 1:** KAP scores and distribution of patients with hypertrophic cardiomyopathy.

Project	Score	Distributed		
		Excellent	Medium	Difference
Knowledge	7.91 ± 3.14	15 (7.77)	104 (53.89)	74 (38.34)
Manner	34.73 ± 3.16	25 (12.95)	168 (87.05)	0 (0.00)
Behavior	39.44 ± 3.95	123 (63.73)	63 (32.64)	4 (2.07)
Total score	82.08 ± 7.84	7 (3.63)	136 (70.47)	50 (25.91)

## 2 Materials and methods

### 2.1 General information

This study used a convenient sampling method and selected 193 patients with hypertrophic cardiomyopathy who were treated in our hospital from June 2022 to June 2024 as the research sample. Among them, 125 were male (accounting for 64.77%) and 68 were female (accounting for 35.23%). Inclusion criteria included: (1) meeting the diagnostic criteria for hypertrophic cardiomyopathy; (2) being conscious and able to express personal opinions clearly and accurately. Exclusion criteria were: (1) having hearing or speech impairment and being unable to complete the communication required for the study; (2) having impaired cognitive function and being unable to participate in the study effectively; (3) suffering from other blood system diseases or metabolic disorders; (4) Omitting, missing or refusing to fill in the questionnaire. This study protocol has been approved by the Medical Ethics Committee of our hospital, and all participants signed the informed consent form after fully understanding the purpose and process of the study and voluntarily joined this study.

### 2.2 Methods

#### 2.2.1 Questionnaire

Based on the KAP theoretical framework and cardiac rehabilitation guidelines, combined with literature review, this study designed a questionnaire covering four dimensions of basic patient information, knowledge, attitude and practice. Basic patient information includes: personal information, economic status, living habits, medical history, treatment details, etc.; 12 knowledge items on hypertrophic cardiomyopathy, scoring is based on the correctness of the answer, with 1 point for a correct answer and 0 points for an incorrect or uncertain answer; 9 attitude items to assess patients' attitudes towards monitoring symptoms, health checks, drug control, postoperative follow-up and cost impact, using Likert 5-level scoring, with options ranging from “strongly disagree, disagree, neutral, agree, strongly agree”, and corresponding scores increasing from 1 to 5. The higher the total score, the more positive the patient's attitude; 9 behavioral items to examine patients' review frequency, treatment compliance, lifestyle adjustments and disease management practices, using Likert 5-level scoring, with options set to “never, occasionally, sometimes, often, always”, and corresponding scores of 1–5. The higher the total score, the more in line with the recommended standards the patient's behavior. The total score and score ratio (actual score/maximum possible score × 100%) were used for evaluation, and the score ratio >85% was excellent, 60%-85% was moderate, and <60% was poor. The reliability coefficient of the questionnaire was 0.917, and the Cronbach's *α* of each dimension was: knowledge 0.858, attitude 0.954, behavior 0.763, and the overall α coefficient was 0.873, showing high reliability and validity.

#### 2.2.2 Survey method

The researchers conducted an online questionnaire survey through the “Wenjuxing” platform. After introducing the purpose and process of the study and obtaining informed consent, the participants completed the questionnaire independently, and each person was limited to submitting it once. For participants who cannot complete it independently, the investigator will assist them to complete it. The criteria for invalid questionnaires are: more than a quarter of the basic information is not answered, all questions are the same or have obvious regularity, and there is a logical contradiction between the options. According to the international scale design principles, the sample size is set at 5–10 times the number of items plus a 20% safety margin, and the minimum sample size is 180. 200 questionnaires were actually distributed, 193 were collected, and the recovery rate was 96.5%.

### 2.3 Statistical analysis

After the data were exported from the Wenjuanxing platform, they were verified and confirmed to be correct, and SPSS 23.0 software was used for statistical analysis. Use the Shapiro Wilk method for normal distribution testing. Count data were presented in the form of frequency (percentage/%); for measurement data that conformed to the normal distribution, the mean ± standard deviation was used for description. For the comparison of differences between the two groups, the independent sample *t-*test was used; and for the difference analysis of three or more groups, the one-way analysis of variance was used. In this study, the rehabilitation knowledge, beliefs and behavior scores of patients with hypertrophic cardiomyopathy were used as dependent variables, and the factors with statistical significance in the univariate analysis were used as independent variables. The multivariate linear regression model was used to explore the factors affecting the level of patients' rehabilitation knowledge, beliefs and behaviors.

## 3 Results

### 3.1 Scores of rehabilitation knowledge, attitude and practice in patients with hypertrophic cardiomyopathy

The KAP questionnaire score of 193 patients with HCM was (82.08 ± 7.84) points, with a score rate of 80.47%. The scores of each dimension of KAP were (7.91 ± 3.14) points, (34.73 ± 3.16) points, and (39.44 ± 3.95) points, with corresponding score rates of 65.93%, 77.18%, and 87.63%.

Twenty two Scores of rehabilitation knowledge, attitude and practice in patients with hypertrophic cardiomyopathy with different characteristics univariate analysis showed that age, place of residence, education level, work status, and monthly per capita income may be factors affecting the rehabilitation knowledge score of patients with hypertrophic cardiomyopathy (all *P* < 0.05). Place of residence, education level, work status, monthly per capita income, smoking, and drinking may be factors affecting the rehabilitation belief score of patients with hypertrophic cardiomyopathy (all *P* < 0.05). Gender, education level, and monthly per capita income may be factors affecting the rehabilitation behavior score of patients with hypertrophic cardiomyopathy (all *P* < 0.05). Gender, place of residence, education level, work status, monthly per capita income, and smoking may be factors affecting the total rehabilitation score of patients with hypertrophic cardiomyopathy (all *P* < 0.05) see Table 2.

**Table 2:** Comparison of scores on the rehabilitation knowledge, attitude and practice questionnaire in patients with hypertrophic cardiomyopathy with different characteristics.

Factor	Number of cases (%)	Knowledge score	Attitude score	Behavioral scores	Total score of knowledge, belief and action
Gender					
Male	125 (64.77)	7.88 ± 3.34	34.68 ± 3.16	39.62 ± 3.87	82.18 ± 7.76
Female	68 (35.23)	7.97 ± 2.75	34.82 ± 3.16	39.09 ± 4.08	81.88 ± 7.98
*t*		0.190	0.300	3.075	2.240
*P*		0.849	0.765	0.002	0.026
Age (years)					
<60	143 (74.09)	8.18 ± 3.13	34.62 ± 3.30	39.38 ± 4.04	82.18 ± 8.16
≥60	50 (25.91)	7.14 ± 3.05	35.04 ± 2.68	39.60 ± 3.68	81.78 ± 6.85
*t*		2.028	0.802	0.341	0.310
*P*		0.044	0.424	0.734	0.757
Place of residence					
Rural	42 (21.76)	6.43 ± 3.13	33.57 ± 3.04	38.60 ± 4.33	78.60 ± 7.82
Town	145 (75.13)	8.27 ± 2.99	35.01 ± 3.10	39.68 ± 3.75	82.96 ± 7.46
Suburban/urban-rural fringe	6 (3.11)	9.67 ± 3.45	36.17 ± 3.34	39.33 ± 5.06	85.17 ± 9.51
*F*		6.912	4.110	1.230	5.768
*P*		0.001	0.018	0.294	0.004
Education					
Primary school and below	15 (7.77)	5.20 ± 3.15	34.93 ± 2.98	38.00 ± 4.90	78.13 ± 7.68
Junior high school	40 (20.73)	6.50 ± 3.29	32.50 ± 2.86	38.73 ± 4.66	77.73 ± 8.01
High school/technical secondary school	37 (19.17)	7.22 ± 2.85	34.51 ± 2.66	38.49 ± 3.70	80.22 ± 7.27
College/Bachelor's degree	93 (48.19)	8.99 ± 2.63	35.57 ± 3.04	40.24 ± 3.27	84.80 ± 6.74
Master degree and above	8 (4.15)	10.75 ± 0.66	36.75 ± 2.44	40.75 ± 3.99	88.25 ± 4.05
*F*		11.705	8.655	2.596	10.175
*P*		<0.001	<0.001	0.038	<0.001
Working status					
Working	77 (39.90)	8.94 ± 2.64	35.68 ± 3.20	39.83 ± 4.06	84.44 ± 7.70
Unemployment	11 (5.70)	6.82 ± 3.74	33.27 ± 2.96	37.27 ± 4.84	77.36 ± 7.01
Retire	50 (25.91)	7.46 ± 3.09	34.68 ± 2.89	39.34 ± 3.36	81.48 ± 7.35
Self-employed	13 (6.74)	5.62 ± 3.91	33.61 ± 3.03	40.69 ± 3.67	79.92 ± 7.34
Housewife	15 (7.77)	6.20 ± 2.97	34.00 ± 2.85	38.47 ± 3.91	78.67 ± 7.96
Other	27 (13.99)	8.33 ± 2.62	33.67 ± 2.96	39.30 ± 3.96	81.30 ± 7.35
*F*		4.908	3.090	1.270	3.233
*P*		<0.001	0.011	0.279	0.008
Average monthly income					
<2,000	30 (15.54)	6.03 ± 3.18	33.63 ± 3.03	37.13 ± 5.02	76.80 ± 8.19
2,000–5,000	56 (29.02)	7.48 ± 2.95	34.48 ± 3.18	40.05 ± 3.10	82.02 ± 7.18
5,000–10,000	54 (27.98)	8.35 ± 2.95	35.46 ± 2.66	39.98 ± 3.51	83.80 ± 6.91
10,000–20,000	15 (7.77)	9.93 ± 1.53	36.33 ± 2.91	41.40 ± 2.60	87.67 ± 4.56
>20,000	12 (6.22)	10.08 ± 1.75	35.92 ± 2.50	40.67 ± 3.09	86.67 ± 4.33
Unwilling to disclose	26 (13.47)	7.92 ± 3.57	33.54 ± 3.61	37.92 ± 4.42	79.38 ± 8.67
*F*		5.500	3.409	4.624	7.155
*P*		<0.001	0.006	0.001	<0.001
Marital status					
Unmarried	17 (8.81)	9.41 ± 1.82	35.06 ± 4.43	40.53 ± 4.03	85.00 ± 8.66
Married	162 (83.94)	7.75 ± 3.24	34.69 ± 2.94	39.40 ± 3.83	81.84 ± 7.52
Divorce	8 (4.15)	8.75 ± 2.05	34.38 ± 3.20	37.88 ± 5.42	81.00 ± 9.63
Widowed	6 (3.11)	6.83 ± 3.34	35.33 ± 4.27	39.50 ± 3.86	81.67 ± 9.41
*F*		1.868	0.173	0.849	0.887
*P*		0.136	0.915	0.469	0.449
Smoking					
Never smoke	104 (53.89)	8.13 ± 2.83	35.14 ± 3.08	39.82 ± 3.83	83.10 ± 7.57
Former smoking	55 (28.50)	7.25 ± 3.40	33.51 ± 2.55	38.93 ± 4.25	79.69 ± 7.39
Still smoking now	34 (17.62)	8.29 ± 3.47	35.44 ± 3.69	39.09 ± 3.67	82.82 ± 8.51
*F*		1.718	6.146	1.065	3.660
*P*		0.182	0.003	0.347	0.028
Drinking					
Never drink alcohol	90 (46.63)	7.92 ± 2.84	35.03 ± 3.26	39.71 ± 3.84	82.67 ± 7.88
Drinking alcohol before	79 (40.93)	7.86 ± 3.22	34.10 ± 2.93	39.14 ± 4.09	81.10 ± 7.71
Still drinking	24 (12.44)	8.04 ± 3.88	35.67 ± 3.12	39.38 ± 3.82	83.08 ± 7.77
*F*		0.031	3.084	0.438	1.059
*P*		0.970	0.048	0.646	0.349
Health insurance					
No medical insurance	0 (0.00)	–	–	–	–
Have social health insurance (social insurance)	161 (83.42)	7.88 ± 3.11	34.78 ± 3.15	39.45 ± 3.83	82.11 ± 7.64
There are social medical insurance (social insurance) and other commercial insurance	32 (16.58)	8.09 ± 3.30	34.50 ± 3.21	39.34 ± 4.55	81.94 ± 8.78
*t*		0.357	0.450	0.143	0.110
*P*		0.722	0.653	0.887	0.912
Diagnosis time					
Less than one year	62 (32.12)	7.40 ± 3.59	34.58 ± 3.38	39.77 ± 3.97	81.76 ± 8.29
1–3 years	37 (19.17)	7.89 ± 3.07	34.59 ± 2.82	39.08 ± 4.52	81.57 ± 8.38
3–5 years	18 (9.33)	8.17 ± 3.20	35.67 ± 3.30	39.89 ± 4.03	83.72 ± 8.56
5+ years	76 (39.38)	8.28 ± 2.69	34.70 ± 3.05	39.22 ± 3.57	82.20 ± 6.90
*F*		0.915	0.592	0.396	0.351
*P*		0.435	0.621	0.756	0.788
Diagnosis type					
Non-obstructive	119 (61.66)	8.16 ± 3.02	34.92 ± 3.25	39.32 ± 4.06	82.39 ± 8.31
Obstructive	74 (38.34)	7.51 ± 3.30	34.43 ± 2.98	39.62 ± 3.78	81.57 ± 6.99
*t*		1.388	1.031	0.514	0.710
*P*		0.167	0.904	0.608	0.479
Combined with other underlying or chronic diseases					
Hypertension	73 (37.82)	7.84 ± 2.90	35.11 ± 2.95	39.53 ± 3.62	82.48 ± 6.51
High cholesterol	57 (29.53)	8.09 ± 2.92	35.30 ± 2.99	39.51 ± 3.66	82.89 ± 6.55
Diabetes	25 (12.95)	8.72 ± 2.52	35.16 ± 2.38	39.28 ± 4.03	83.16 ± 6.32
Coronary heart disease	20 (10.36)	6.55 ± 3.29	34.30 ± 2.45	39.35 ± 4.21	80.20 ± 6.33
Tumor	4 (2.07)	7.50 ± 3.77	37.00 ± 3.08	38.25 ± 6.30	82.75 ± 12.09
Other	19 (9.84)	7.00 ± 2.81	35.32 ± 3.13	39.32 ± 3.48	81.63 ± 6.59
None	78 (40.41)	8.10 ± 3.30	34.28 ± 3.46	38.83 ± 4.66	81.22 ± 9.44
*F*		1.322	1.281	0.272	0.627
*P*		0.248	0.266	0.950	0.709
Whether there is a history of heart surgery					
Yes	28 (14.51)	8.00 ± 2.87	34.11 ± 3.02	40.29 ± 3.15	82.39 ± 6.81
No	165 (85.49)	7.89 ± 3.19	34.84 ± 3.17	39.29 ± 4.06	82.02 ± 8.00
*t*		0.160	1.127	1.229	0.229
*P*		0.873	0.210	0.220	0.819
Medications currently in use					
Metoprolol/Atenolol/Bisoprolol	143 (74.09)	8.30 ± 2.79	35.01 ± 3.20	39.80 ± 3.77	83.10 ± 7.42
Diuretics	62 (31.12)	8.34 ± 2.83	35.34 ± 2.98	39.95 ± 3.44	83.63 ± 6.79
Anticoagulants	26 (13.47)	7.88 ± 2.90	34.73 ± 3.18	39.35 ± 3.81	81.96 ± 6.91
Sacubitril/Valsartan/Captopril/Irbesartan	52 (26.94)	7.48 ± 3.55	34.67 ± 3.41	39.38 ± 4.46	81.54 ± 8.54
Spironolactone	31 (16.06)	6.87 ± 3.41	34.03 ± 3.28	38.39 ± 4.54	79.29 ± 8.83
Empagliflozin/Dapagliflozin	29 (15.03)	7.83 ± 3.13	34.66 ± 2.99	39.10 ± 3.95	81.59 ± 7.85
Other	51 (26.42)	7.88 ± 3.45	34.41 ± 2.86	39.98 ± 3.27	82.27 ± 6.91
*F*		1.298	0.855	0.858	1.520
*P*		0.257	0.528	0.526	0.170

### 3.3 Multivariate linear regression analysis of rehabilitation knowledge, attitude and practice in patients with hypertrophic cardiomyopathy

Multivariate linear regression analysis was performed on the factors with statistical significance in the univariate analysis. The results showed that education level, work status and monthly average income were important factors affecting the scores of patients' rehabilitation knowledge, attitudes and behaviors (*P* < 0.05), as shown in Table 3.

**Table 3:** Multivariate linear regression analysis of factors affecting rehabilitation knowledge, attitudes and behaviors in patients with hypertrophic cardiomyopathy.

Project		*B*	Standard error	Standardized coefficient	*t*	*P*
Knowledge	Constant	4.955	1.472	–	3.367	0.001
	Age	−0.035	0.016	−0.152	−2.181	0.03 0
	Place of residence	0.635	0.53 0	0.093	1.197	0.233
	Education	0.995	0.251	0.336	3.972	<0.001
	Working status	0.017	0.127	0.01 0	0.137	0.891
	Average monthly income	0.118	0.141	0.059	0.835	0.405
Belief	Constant	33.191	1.445	–	22.969	<0.001
	Place of residence	0.327	0.564	0.048	0.580	0.562
	Education	0.743	0.258	0.249	2.874	0.005
	Working status	0.943	0.284	0.206	3.325	0.011
	Average monthly income	0.563	0.217	0.473	2.594	<0.001
	Smoking	−0.068	0.354	−0.016	−0.191	0.849
	Drinking	−0.206	0.412	−0.045	−0.500	0.618
Behavior	Constant	37.662	1.283	–	29.346	0.000
	Gender	−0.370	0.591	−0.045	−0.626	0.532
	Education	0.806	0.284	0.216	2.835	0.005
	Average monthly income	1.246	0.251	0.269	4.961	<0.001
Total score	Constant	74.022	4.007	–	18.472	<0.001
	Gender	−0.477	1.419	−0.029	−0.336	0.737
	Place of residence	0.903	1.363	0.053	0.663	0.508
	Education	2.729	0.625	0.369	4.366	<0.001
	Working status	2.383	0.756	0.281	3.152	0.003
	Average monthly income	2.790	0.651	0.390	4.286	<0.001
	Smoking	−0.818	0.859	−0.08	−0.952	0.342

## 4 Discussions

HCM is characterized by asymmetric myocardial thickening and has complex and diverse pathophysiological mechanisms. It is the leading cause of sudden cardiac death in young people and athletes (11, 12). In the field of public health, knowledge, attitude and practice (KAP) research has been widely used to assess the cognition, attitude and behavior of different populations towards health issues (13). This study found that HCM patients scored above average in the three dimensions of knowledge, attitude and behavior. The behavior dimension scored the highest, with 63.73% of patients reaching the excellent standard; while the knowledge dimension scored the lowest, with 38.34% of patients being rated as poor. The degree of mastery of health-related knowledge directly affects individual attitudes and can trigger changes in behavior patterns (14). Therefore, improving the understanding of HCM-related knowledge is crucial to changing health behaviors and lifestyles. The results showed that HCM patients had insufficient knowledge of the causes and treatments of the disease, resulting in low scores in this dimension. This phenomenon may be related to the effectiveness of information dissemination and education level, and also reflects the limitations of current patient health education in resource allocation and management strategies (15). Improving disease cognition in HCM patients is an important prerequisite for improving their health management.

Analyzing the rehabilitation knowledge, belief, and behavior scores of HCM patients with different characteristics, it was found that factors such as age, place of residence, education, working status, and monthly per capita income significantly affected the patients’ knowledge, belief, and behavior scores (*P* < 0.05). This result is consistent with the results of Machaalani M et al. Research is consistent (16). Lower socioeconomic status and inadequate social support may limit a patient's ability to access and understand medical information. Older patients may have difficulty absorbing new health knowledge effectively due to cognitive decline or impairment in the use of information technology. In addition, urban residents perform better in health literacy because they have easier access to medical services and information resources (17). Improvements in education levels are closely related to increases in health literacy (18), while increases in income help obtain more medical care resources and promote healthy behaviors (19). A healthy lifestyle is associated with lower mortality and reduced risk of cardiovascular disease (20). Smoking significantly increases the risk of acute and chronic cardiovascular disease, but its negative effects can be quickly alleviated after quitting smoking (21). For the behavioral scores of HCM patients, gender, education level and monthly per capita income showed significant effects (*P* < 0.05). Female patients may face more challenges when implementing rehabilitation programs, such as family responsibilities and social role restrictions, which not only increases their psychological vulnerability, but may also lead to poorer physical recovery than male patients and increase the risk of emotional disorders such as anxiety and depression (22). Perception of cardiovascular disease risk is closely related to education level, and higher education levels are usually associated with better socioeconomic status and greater access to medical care (23). Studies in Poland have confirmed the association between low education and socioeconomic status and the risk of cardiovascular disease (24, 25). Low educational levels and financial difficulties often limit patients' ability to engage in healthy behaviors.

Multivariate linear regression analysis showed that education level, work status and average monthly income were independent factors affecting the knowledge, attitude and behavior scores of HCM patients. A high education level is closely related to health behavior (26), and individuals with higher education levels are more likely to understand and adopt disease prevention recommendations. Education not only shapes the occupational environment and opportunities, but also affects the living environment and psychological exposure related to long-term health. By improving the level of education, individuals can enhance their economic ability, obtain high-quality medical services, and enjoy direct and long-term material advantages, thereby strengthening the positive association between income and life expectancy (27). Previous studies have confirmed socioeconomic differences in cardiovascular disease (28, 29), and individual socioeconomic status is negatively correlated with cardiovascular disease risk (30), highlighting the decisive role of economic status on health.

In summary, the results of this study provide a new perspective for understanding the social determinants of HCM and lay the foundation for developing comprehensive prevention and intervention measures. Future research should design and implement targeted intervention strategies based on the current rehabilitation cognition, attitude, behavioral status, and related influencing factors of such patients, optimize their rehabilitation cognition, attitude, and behavior, thereby improving their quality of life and optimizing public health services.

## Data Availability

The datasets presented in this study can be found in online repositories. The names of the repository/repositories and accession number(s) can be found in the article/Supplementary Material.
